# Supplementary material for: Socio-Demographic Correlates of Total and Domain-Specific Sedentary Behavior in Latin America: A Population-Based Study
Source: Int J Environ Res Public Health. 2020 Aug 3;17(15):5587. doi: 10.3390/ijerph17155587 (PMC7432879; doi:10.3390/ijerph17155587)
Supplement: Supplementary file 1 [file ijerph-17-05587-s001.pdf]

International Journal of
Environmental Research and Public Health

MDPI

Supplementary Materials: Socio-Demographic Correlates of Total and Domain-Specific Sedentary Behavior in Latin America: A Population-Based Study

Gerson Luis de Moraes Ferrari [1,*], André Oliveira Werneck [2], Danilo Rodrigues da Silva [3], Irina Kovalskys [4], Georgina Gómez [5], Attilio Rigotti [6], Lilia Yadira Cortés Sanabria [7], Martha Cecilia Yépez García [8], Rossina G. Pareja [9], Marianella Herrera-Cuenca [10], Ioná Zalcman Zimberg [11], Viviana Guajardo [4], Michael Pratt [12], Cristian Cofre Bolados [1], Rodrigo Fuentes Kloss [1], Scott Rollo [13,14], Mauro Fisberg [15,16] and on behalf of the ELANS Study Group ‡

Table S1. Min/day (mean [95% CI]) of sedentary behavior by country for specific-domains.

Country	Computer use at Home	Videogame Use	Reading	Socializing or Listening to Music	Talking on the Telephone	Watching TV	Riding in an Automobile
Argentina	96.7 (89.2; 105.3)	75.8 (63.6; 89.5)	64.4 (58.8; 70.7)	109.5 (103.3; 116.8)	47.5 (43.8; 51.1)	138.8 (132.8; 145.4)	83.3 (72.9; 94.2)
Brazil	156.5 (141.6; 170.8)	135.8 (109.8; 164.6)	83.3 (73.4; 94.9)	109.3 (102.7; 116.9)	51.2 (46.8; 56.2)	172.3 (162.7; 184.3)	80.4 (74.2; 87.8)
Chile	105.5 (96.0; 115.0)	74.8 (60.3; 90.9)	47.9 (42.6; 53.9)	73.1 (67.6; 78.7)	35.5 (31.1; 39.8)	114.9 (109.3; 120.7)	63.9 (56.4; 71.9)
Colombia	115.0 (103.5; 126.7)	87.8 (74.7; 103.9)	56.3 (51.2; 62.1)	102.3 (95.4; 109.7)	53.5 (48.2; 58.5)	139.8 (132.9; 147.9)	102.8 (88.2; 119.0)
Costa Rica	161.6 (138.0; 187.3)	144.4 (113.1; 185.5)	86.3 (72.4; 103.6)	139.5 (127.6; 153.5)	53.2 (46.8; 60.9)	220.3 (201.2; 239.4)	150.5 (123.5; 178.8)
Ecuador	73.6 (67.1; 81.1)	69.8 (59.6; 81.6)	38.1 (33.9; 42.8)	63.9 (59.3; 68.6)	30.4 (27.3; 34.2)	101.3 (96.6; 106.2)	80.2 (70.6; 90.3)
Peru	105.6 (94.4; 118.8)	88.1 (73.8; 104.2)	44.7 (41.3; 48.2)	74.9 (70.0; 80.3)	53.1 (48.6; 57.5)	145.0 (137.5; 153.4)	103.2 (80.7; 127.9)
Venezuela	83.1 (76.7; 90.0)	72.3 (58.3; 87.3)	39.5 (36.4; 43.5)	78.4 (73.9; 83.3)	34.7 (31.4; 38.3)	120.0 (115.5; 124.3)	95.5 (84.6; 106.8)
Overall	115.1 (110.5; 119.9)	96.4 (89.1; 104.1)	58.2 (55.4; 60.9)	95.5 (93.0; 98.2)	46.4 (44.6; 48.3)	146.3 (142.8; 149.8)	90.9 (86.4; 95.3)
